# The 2016 Bioinformatics Open Source Conference (BOSC)

**DOI:** 10.12688/f1000research.9663.1

**Published:** 2016-10-06

**Authors:** Nomi L. Harris, Peter J.A. Cock, Brad Chapman, Christopher J. Fields, Karsten Hokamp, Hilmar Lapp, Monica Muñoz-Torres, Heather Wiencko

**Affiliations:** 1 Lawrence Berkeley National Laboratory, Berkeley, CA, 94720, USA; 2 The James Hutton Institute, Dundee, DD2 5DA, UK; 3 Bioinformatics Core, Harvard T.H. Chan School of Public Health, Boston, MA, 02115, USA; 4 High Performance Computing in Biology Group, Carver Biotechnology Center, University of Illinois Urbana-Champaign, Urbana, IL, 61801, USA; 5 Smurfit Institute of Genetics, Trinity College Dublin, Dublin 2, Ireland; 6 Center for Genomic and Computational Biology, Duke University, Durham, NC, 27708, USA; 7 Plusvital, NovaUCD, Belfield, Dublin 4, Ireland

**Keywords:** Bioinformatics, open source, open science

## Abstract

**Message from the ISCB:**

The Bioinformatics Open Source Conference (BOSC) is a yearly meeting organized by the Open Bioinformatics Foundation (OBF), a non-profit group dedicated to promoting the practice and philosophy of Open Source software development and Open Science within the biological research community. BOSC has been run since 2000 as a two-day Special Interest Group (SIG) before the annual ISMB conference. The 17th annual BOSC ( http://www.open-bio.org/wiki/BOSC_2016) took place in Orlando, Florida in July 2016. As in previous years, the conference was preceded by a two-day collaborative coding event open to the bioinformatics community. The conference brought together nearly 100 bioinformatics researchers, developers and users of open source software to interact and share ideas about standards, bioinformatics software development, and open and reproducible science.

## Message from the ISCB

## Introduction

BOSC is a “community of communities,” bringing together participants from many open source projects and organizations to learn from each other and to provide opportunities for forming synergistic alliances to tackle bigger problems together. The conference includes two days of talks, posters, a panel discussion, and Birds of a Feather interest groups (BOFs). Session topics this year included Data Science; Standards and Interoperability; Open Science and Reproducibility; Workflows; Developer Tools and Libraries; and a session for late-breaking lightning talks. BOSC’s broad spectrum of topics results in a highly diverse mix of presentations, covering many different resources and providing something of interest to all attendees, regardless of their background or expertise.

The complete program for BOSC 2016 is available at http://www.open-bio.org/wiki/BOSC_2016. Links to articles, blog posts, and Twitter summaries of the conference can be found there as well. Most of the slides and posters from BOSC 2016 are hosted on our *F1000Research* channel ( http://f1000research.com/channels/BOSC), and videos of the presentations can be found on our YouTube channel.

The city hosting the conference this year, Orlando, was in the news recently for a tragic mass shooting at the Pulse nightclub, a popular spot for the local LGBTQ community. The BOSC organizers, led by Mónica Muñoz-Torres, acknowledged and paid tribute to the victims of that massacre, both explicitly (at the beginning and end of the meeting) and implicitly with a new rainbow-striped version of our familiar pear logo. Dr. Muñoz-Torres also organized a BOF on activism in the professional world (see http://www.open-bio.org/bosc2016/MMT-Intro-Summary-BoF-BOSC2016.pdf), which identified ways to help the Pulse victims’ families and to make BOSC and other meetings more accessible and welcoming to a wide and inclusive community.

**Figure 1.**
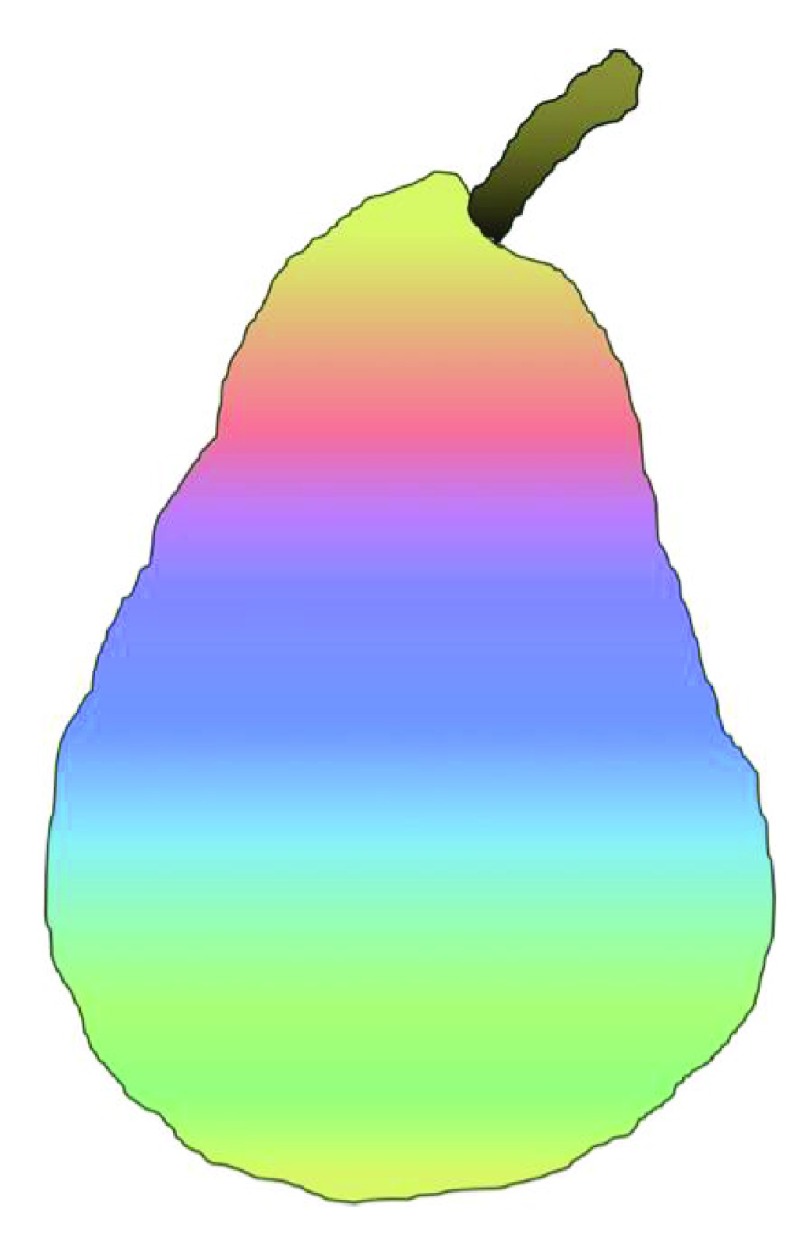
BOSC 2016 logo. The new rainbow-striped version of the familiar BOSC pear symbolizes our “community of communities” and commitment to be inclusive of traditionally underrepresented and marginalized groups, including the LGBTQ community, which was specifically targeted in Orlando’s Pulse nightclub.

## Panel

In recent years, BOSC has included a panel discussion that offers its attendees the chance to engage in conversation with the panelists and each other on a subject that presents a contemporary and cross-cutting challenge for open source bioinformatics. This year’s panel focused on how to grow and sustain open source communities. The panelists represented various thriving projects and initiatives active in the open source space: Abigail Cabunoc Mayes (Mozilla Science Lab), Bastian Greshake (openSNP), Jamie Whitacre (Project Jupyter), John Chilton (Galaxy), Natasha Wood (Cape Unseminars in Bioinformatics), and panel chair Mónica Muñoz-Torres (Berkeley Bioinformatics Open-Source Projects).

**Figure 2.**
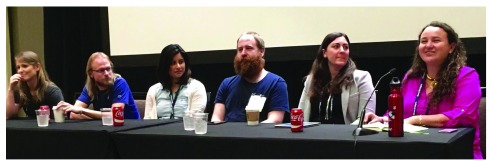
Panelists Natasha Wood, Bastian Greshake, Abigail Cabunoc Mayes, John Chilton, and Jamie Whitacre with panel chair Mónica Muñoz-Torres.

Questions addressed to the panel included:

- Why invest in community development?
- How can a project build bridges between communities but still maintain its identity and ownership?
- What is the value of empathy in the community building process?
- How best to recognize contributions from community members?
- What is the best strategy for dissemination and growth?
- When is a project large enough to recruit people focused on community development?

The perspectives voiced by panel members highlighted issues that tend to receive less attention but that can affect the sustainability of an open source community, such as the importance of having a clearly articulated mission; recognizing and even rewarding contributions (e.g., with contributor badges); and providing newcomers with low-barrier opportunities to join (e.g., by paying attention to novice-friendly documentation, and by splitting up larger codebases). More information about the panel can be found among the reactions posted on Twitter (see https://storify.com/HLWiencko/bosc2016-panel and https://smallchangebio.wordpress.com/2016/07/08/bosc2016day1b/).

## Keynote talks

**Figure 3.**
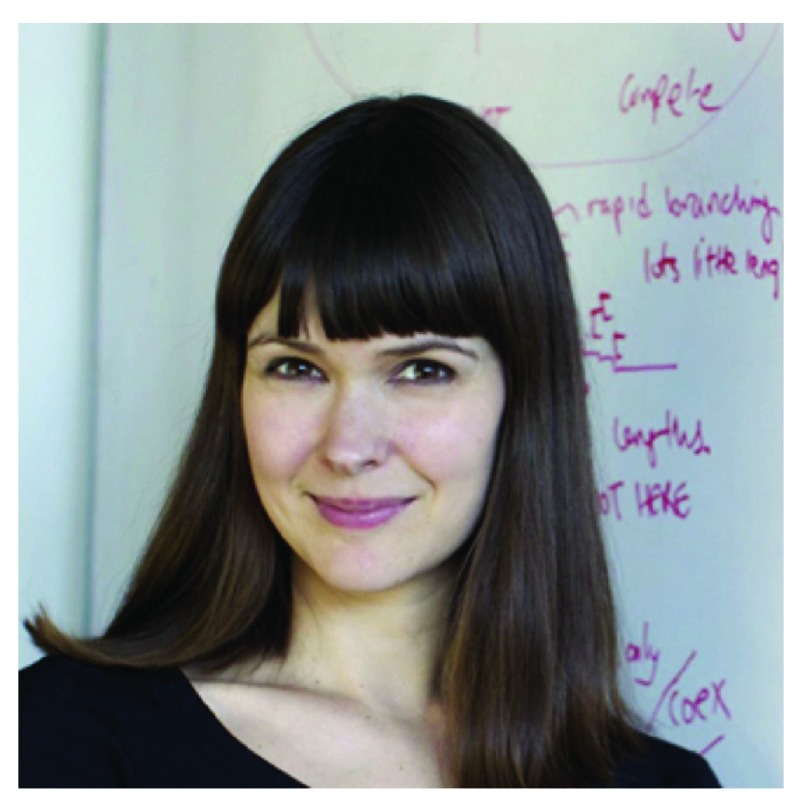
Keynote speaker Jennifer Gardy.

Keynote talks by researchers who are influential in some aspect of open source bioinformatics are a popular part of BOSC. Past speakers include bioinformatics luminaries such as Phillip Bourne, Sean Eddy, Jonathan Eisen, Carole Goble and Ewan Birney. This year’s keynote speakers were Jennifer Gardy and Steven Salzberg.

Jennifer Gardy is known both as a scientist (she’s a bioinformatics and public health professor at the University of British Columbia and a senior scientist at the British Columbia Centre for Disease Control) and as a science communicator who appears regularly on science TV shows including Project X and the Discovery Channel’s Daily Planet science show. Her keynote talk, entitled “The open-source outbreak: can data prevent the next pandemic?” vividly demonstrated how open data, open bioinformatics analysis and crowdsourcing can help us understand and contain infectious disease pandemics more quickly and efficiently.

**Figure 4.**
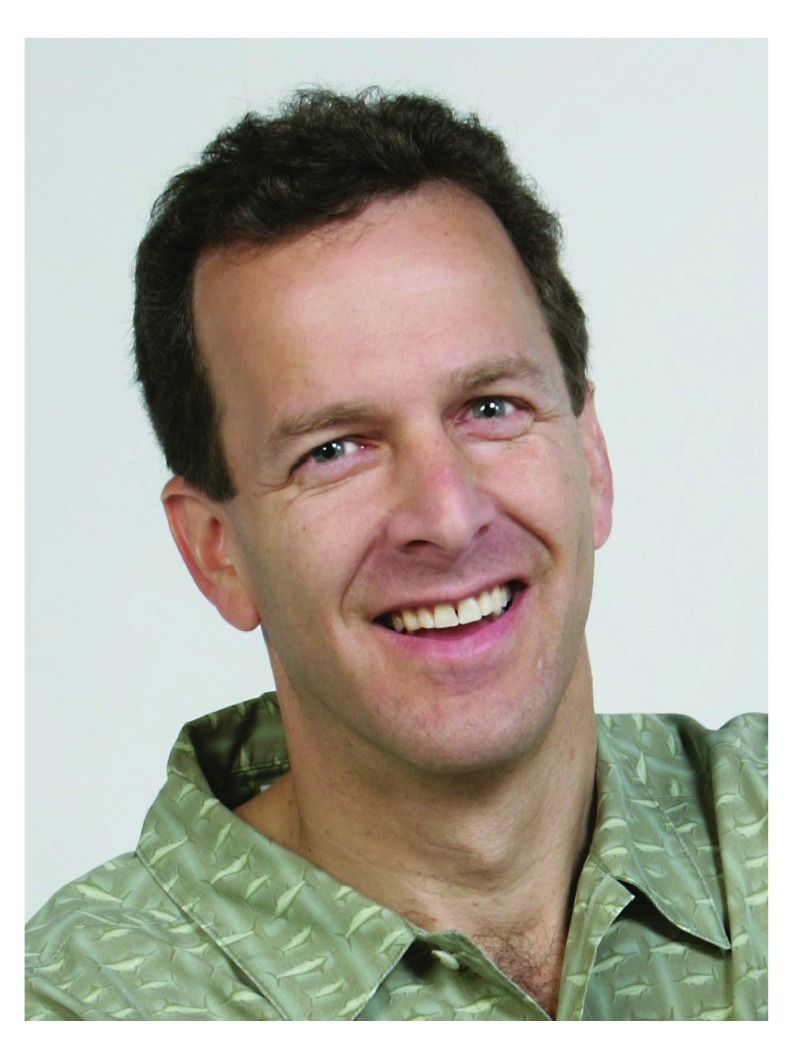
Keynote speaker Steven Salzberg.

Our second keynote speaker, Steven Salzberg, is a long-time pillar of the open bioinformatics community. He was awarded the Benjamin Franklin Award for Open Access in the Life Sciences in 2013. Dr. Salzberg’s talk touched on several types of openness: open source, open data and open access publishing. He discussed how openness can accelerate and improve the quality of scientific research, while also acknowledging some of the challenges openness presents, such as receiving credit for your work, retaining some control over authorship and protecting sensitive human data.

## Codefest 2016

Every year since 2010, a collaborative community development event called Codefest has been held the two days before BOSC. The event is open to anyone interested, has no registration fee and provides a venue for open source bioinformatics developers to meet in person to work on or plan joint projects. Codefest 2016 ( http://www.open-bio.org/wiki/Codefest_2016) was hosted by the FamiLAB community makerspace in Orlando. Forty community members worked on projects including improvements to the Common Workflow Language, integration between the ADAM and Nextflow analysis environments, additions to the MultiQC reporting framework, and enhancements to existing Open Bioinformatics projects including BioPerl, BioJava and Biopython,

## Sponsorships

Sponsorships allow BOSC to offer free registration to some speakers who would otherwise find it financially difficult to attend BOSC. We are grateful to returning sponsor Curoverse (the team behind the open source platform Arvados) for sponsoring both BOSC 2016 and Codefest, and to new sponsor PLOS Computational Biology for contributing to Codefest.

